# Digital Pathology Scoring of Immunohistochemical Staining Reliably Identifies Prognostic Markers and Anatomical Associations in a Large Cohort of Oral Cancers

**DOI:** 10.3389/fonc.2021.712944

**Published:** 2021-07-29

**Authors:** Julius Moratin, Andreas Mock, Sonja Obradovic, Karl Metzger, Christa Flechtenmacher, Karim Zaoui, Stefan Fröhling, Dirk Jäger, Jürgen Krauss, Jürgen Hoffmann, Kolja Freier, Dominik Horn, Jochen Hess, Christian Freudlsperger

**Affiliations:** ^1^Department of Oral and Cranio-Maxillofacial Surgery, University of Heidelberg, Heidelberg, Germany; ^2^Department of Medical Oncology, National Center for Tumor Diseases (NCT) Heidelberg, Heidelberg University Hospital, Heidelberg, Germany; ^3^Department of Translational Medical Oncology, National Center for Tumor Diseases (NCT) Heidelberg, German Cancer Research Center (DKFZ), Heidelberg, Germany; ^4^Institute of Pathology, University of Heidelberg, Heidelberg, Germany; ^5^Department of Otorhinolaryngology, University of Heidelberg, Heidelberg, Germany; ^6^Department of Oral and Maxillofacial Surgery, Saarland University, Homburg, Germany

**Keywords:** immune cells, automated, quantification, biomarker, head and neck squamous cell carcinoma, oral squamous cell carcinoma

## Abstract

Utilizing digital pathology algorithms for the objective quantification of immunohistochemical staining, this study aimed to identify robust prognostic biomarkers for oral cancer. Tissue microarrays with specimens of a large cohort of oral squamous cell carcinoma (n=222) were immunohistochemically stained to determine the expression of PD-L1, EGFR, and COX-2 and the amount of infiltrating NK cells and CD8-positive T cells. Immunoreactivity scores were assessed using both a classical manual scoring procedure and a digital semi-automatic approach using QuPath. Digital scoring was successful in quantifying the expression levels of different prognostic biomarkers (CD8: p<0.001; NK cells: p=0.002, PD-L1: p=0.026) and high levels of concordance with manual scoring results were observed. A combined score integrating EGFR expression, neck node status and immune cell signatures with a significant impact on overall and progression-free survival was identified (p<0.001). These data may contribute to the ongoing research on the identification of reliable and clinically relevant biomarkers for the individualization of primary and adjuvant treatment in oral cancer.

## Introduction

In 2018, head and neck cancer was the seventh most common group of malignant tumors worldwide with approximately 900.000 new cases per year and squamous cell carcinomas arising from the oral mucosa (OSCC) form a major part of this entity (1–3). Metastases and locoregional disease recurrence are the main predicting factors for adverse clinical outcome and OSCC is responsible for about 1.5% of all cancer related deaths in the United States (4, 5). 5-year survival rates have remained at 40-60% over the last decades despite interdisciplinary multi-modal treatment (6–10).

Although, the introduction of immune checkpoint inhibition has brought new therapeutic options for patients with recurrent and/or metastatic head and neck squamous cell carcinoma (HNSCC), accurate biomarkers allowing for distinct risk-stratification and individualization of therapy and follow-up for patients with primary oral cancer are still limited (11, 12). Such markers might help to identify patients at risk of tumor progression, who may benefit from a more intensive interdisciplinary multi-modality therapy. While a plethora of publications reported on potential biomarkers, up to date, only few have been translated into clinical application due to a lack of prognostic relevance and clinical practicality.

A well-established method to identify and evaluate potential biomarkers is immunohistochemical staining of tumor sections to assess the expression pattern of potential candidate proteins. The use of tissue microarrays (TMAs) is a common and efficient technique to investigate expression levels of multiple markers in a large number of different tissue samples (13). The conventional way of manual inspection and counting of stained cells to assess the quantity and quality of potential biomarkers, such as different proteins or tumor-associated immune cells, is strongly observer-dependent and potentially error-prone. The introduction of automated digital image analysis has brought a new technique that may help to standardize and objectify pathological analysis including the assessment of biomarkers (14–16).

Lately, the shift towards a focus on the tumor immune microenvironment (TIME) led to the introduction of new potential biomarkers like immune-checkpoint-proteins (e. g. PD-L1) and tumor-associated immune cells in a variety of tumor entities including HNSCC. In the context of the newly introduced immunotherapy, especially PD-L1 and tumor infiltrating lymphocytes (TILs) are promising candidates with the potential to quantify both relevant aspects, the tumor immunogenicity and patients’ immunological capacity (17–22). Additionally, EGFR and COX-2 have recently been identified as key regulators related to immune phenotypes in head and neck cancer with potential impact on the response to treatment immune checkpoint inhibition (ICI) (23).

Therefore, the purpose of this study was to apply digital pathology algorithms to investigate the expression levels of different potential biomarkers, including EGFR, COX-2 and PD-L1 and the infiltration of cytotoxic TILs like natural killer (NK) cells (defined by NK activation receptor CD335) and CD8+ T cells in tissue specimens of a cohort of 222 OSCCs. Protein expression and immune cell infiltration patterns were then analyzed regarding anatomic distribution and prognostic significance for overall and progression-free survival.

## Materials and Methods

### Patients and Samples

The investigated cohort consisted of 222 patients with primary OSCCs. All patients received surgical treatment at the Department of Oral and Cranio-Maxillofacial Surgery of the University Hospital Heidelberg between the years 2010 and 2016. In case of residual disease, lymph node metastases or histopathological risk factors additional adjuvant radiotherapy or radio-chemotherapy was applied. Written informed consent was obtained from all patients and the study was approved by the ethics committee of the medical faculty of the University of Heidelberg (Ethic vote: S-360/2011). Follow-up data was assessed retrospectively *via* SAP patient management research software (SAP, Walldorf, Germany).

### Tissue Microarray and Histological Slices

All TMAs and histological slices were prepared by the tissue bank of the National Center for Tumor Diseases (NCT) Heidelberg, Germany according to an established protocol as reported earlier (13). Hematoxylin-eosin-stained slides of the prepared tissue samples were examined by an expert pathologist for tumor content. Tumors were then marked to enable the selection of appropriate tissue samples. Via the tissue chip microarray (Beecher Instruments, Sun Prairie, Wisconsin, USA), tissue cores were extracted from the paraffin blocks. After transfer of the tissue cores into a recipient block, paraffin-embedding was used to create TMA blocks and slices were produced with a thickness of 2-3 µm for the staining procedure (Histo Bond, Marienfeld, Germany).

### Immunohistochemistry

TMAs were stained using anti‐PD‐L1 (Cell Signaling Technology, Danvers, Massachusetts, USA), anti-Nkp46/CD335 (Thermo Fisher Scientific, Waltham, Massachusetts, USA), anti-Human CD8, (Clone C8/144B Dako, Agilent Technologies, Santa Clara, USA), anti-EGFR (D38B1, Cell Signaling Technology, Danvers, Massachusetts, USA), and anti-COX2 (SP21, Invitrogen/Thermo Fisher Scientific, Waltham, Massachusetts, USA) monoclonal antibodies, and the DAB Substrate Kit (Vector Laboratories, California, USA) following the manufacturer’s instructions. Afterwards, TMAs were scanned using the Nanozoomer HT Scan System (Hamamatsu Photonics, Japan).

### Manual and Digital Pathology-Based Scoring

The manual scoring procedure was performed with digital scans of the TMAs exclusively using the NDP.view2 software (Hamamatsu Photonics, Hamamatsu, Japan). The immunoreactivity score (IRS) was determined by three independent observers, who assessed the relative amounts of stained cells and the staining intensity. The observers were blinded for the clinical data of the patients included in the study during the scoring procedures. For assessment of tumor cells, an ordinal scale was used based on the number of stained cells and staining intensity (amount of stained cells: 1 = no stained cells, 0%; 2 = < 33%; 3 = 33%-66%; 4 = >66%; staining intensity: 1 = no staining; 2 = low; 3 = medium; 4 = high). Median values of the three observes were used as final score. The two scores were then multiplied to create the final IRS with a range from 1 to 16. For immune cells, an ordinal scale was used based on the number of stained cells (amount of stained cells: 1 = no stained cells, 0%; 2 = < 33%; 3 = 33%-66%; 4 = >66%).

**Figure 1:**
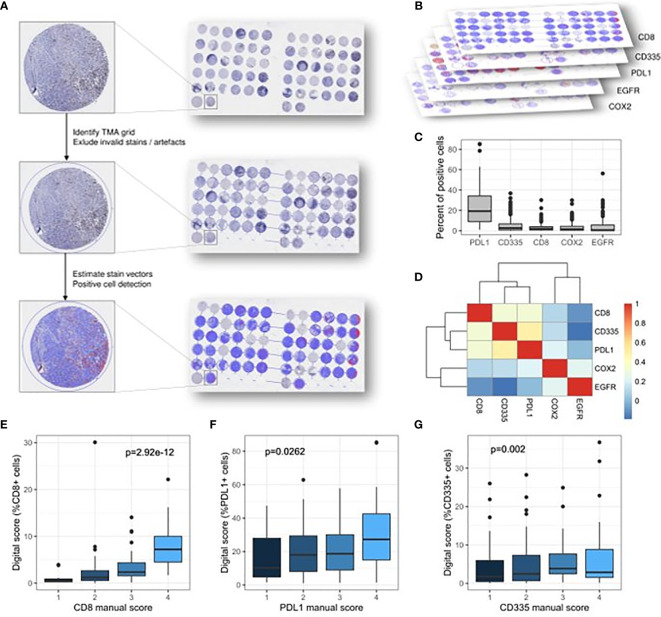
Graphical abstract of the digital scoring procedure principles and the associations between score values obtained by different scoring techniques. **(A)** Identification and evaluation of tissue spots. Detection and quantification of stains. **(B)** Different biomarkers included in the analysis (CD8 cells, CD335/NK cells, PD-L1, EGFR, COX-2). **(C)** Percentage of positive cells for each stain across the samples. **(D)** Heatmap depicting the Spearman’s correlation coefficient between different biomarkers. **(E–G)** Concordance between the classical manual scoring and the digital scoring technique for CD8 cells, CD335/NK cells and PD-L1.

QuPath version v0.2.2 was used for semi-automatic digital quantification of immunohistochemical staining (24). In the first step, the TMA dearrayer function was used to infer the TMA grid. This step was followed by manually excluding invalid samples as well as staining artefacts from further analysis. Next, staining vectors were automatically determined for every TMA slide individually to ensure meaningful quantification between slides. Lastly, the positive cell detection function was used to quantify the number of positive cells for every sample. For every marker, the optimal score compartment was assessed by visual control (nucleus mean for CD8 cells, cytoplasm mean for CD335, EGFR and PD-L1 and cell mean for COX2). **Figure 1A** illustrates the procedure of semi-automatic digital scoring.

### Anatogram of Immunostains

The illustration of the oral cavity was created using the vector-based graphic design software Vectornator X (Linearity GmbH, Karlsruhe). The exact tumor localization in relation to the graphic was determined for each patient using Microsoft Excel (Microsoft, Redmond, Washington). The tumor localization was projected onto the graphic and the various levels of expression (continuous scale) were mapped for each potential biomarker in R (version 4.0.2; www.r-project.org) to illustrate different levels of expression according to the tumor localization in the oral cavity.

### Statistical Analysis

Statistical analyses were performed using Microsoft Excel (Microsoft, Redmond, Washington), SPSS 25 (SPSS for Windows, SPSS, Chicago, IL) and R (version 4.0.2; www.r-project.org).

Demographic, clinical and pathological features of the investigated cohort were analyzed using descriptive statistics. Median values of groups were analyzed using the Kruskal–Wallis one-way analysis of variance. The Wilcoxon rank-sum test was used for pairwise comparison of median values between groups. Correlation of expression levels of different biomarkers were evaluated using Spearman’s correlation coefficient and concordance of values obtained by different scoring procedures was evaluated using linear regression modelling.

The optimal cut-off values to define high- and low-expressing groups in the digital scoring for all investigated biomarkers including the CD8/CD335 ratio were defined in a data-driven approach by finding meaningful local maxima in the distribution of p-values for all cut-offs in the inter-quartile range of expression values (see **Figure 3**).

Survival analysis was performed using the Kaplan-Meier method from date of diagnosis until death, disease recurrence or end of data collection and log-rank testing served to determine differences between the groups. Univariate and multivariate Cox regression models were applied to evaluate the impact of immune cell infiltration and protein expression on overall survival and progression-free survival together with relevant covariates.

A p-value of less than 0.05 was considered statistically significant.

## Results

### Patient Cohort

Overall, tissue samples of 222 patients were included in the analysis. 137 patients (61.7%) were male and 85 (38.3%) were female. The age ranged from 27 to 88 years with a mean age of 64.3 ± 11.1 years. All patients suffered from primary squamous cell carcinoma of the oral cavity and received surgical treatment in the Department of Oral and Cranio-Maxillofacial Surgery of the University of Heidelberg between 2010 and 2016.

112 patients (50.5%) initially presented with early-stage disease (Stage I/II) and 110 (49.5%) with advanced disease (Stage III/IV). Adjuvant radiotherapy or radio-chemotherapy was applied for patients with advanced tumors (Stage III/IV), incomplete tumor resection (R+) or the presence of histopathological risk factors, such as perineural (PN+), lymphatic (L+) or vascular (V+) tumor infiltration. 89 patients (40.1%) received adjuvant treatment including radiotherapy (54 patients) and radio-chemotherapy (35 patients). 47 patients (21.2%) died during follow-up and 47 patients (21.2%) experienced disease recurrence. **Table 1** provides an overview of demographic and clinical features of the patient cohort.

**Table 1:** Demographic, clinical and pathological data of the investigated cohort of 222 oral and oropharyngeal cancers.

Parameter	Number of cases (%)
**Gender**	
Female	85 (38.3)
Male	137 (61.7)
**Age**	
<65 years	111 (50)
>65 years	111 (50)
**T Stage**	
T1	82 (36.9)
T2	72 (32.4)
T3	8 (3.6)
T4	60 (27.1)
**N Stage**	
0	147 (66.2)
1	27 (12.2)
2a	1 (0.5)
2b	28 (12.5)
2c	18 (8.1)
3	1 (0.5)
**M Stage**	
0	222 (100)
1	0
**UICC**	
1	69 (31.1)
2	44 (19.8)
3	23 (10.4)
4	86 (38.7)
**Differentiation Grade**	
1	17 (7.7)
2	153 (68.9)
3	46 (20.7)
Missing	6 (2.7)
**R**	
0	210 (94.6)
1	10 (4.5)
Missing	2 (0.9)
**Location**	
Floor of the mouth	64 (28.8)
Tongue	52 (23.4)
Mandible	70 (31.5)
Maxilla	5 (2.3)
Oropharynx	16 (7.2)
Buccal Plane	14 (6.3)
Lower lip	1 (0.5)
**Recurrence**	
yes	47 (21.2)
no	175 (78.8)

### Digital Pathology Scoring of Immunostaining and Comparison With Manual Scoring

The digital scoring of immunostaining was performed for CD8, CD335, PD-L1, EGFR and COX2 (**Figures 1B, C**). The immunostaining for CD8, CD335 and PD-L1 were also evaluated by manual scoring enabling a comparative analysis between both techniques. No significant correlation could be observed between all markers (**Figure 1D**). The highest concordance was achieved for CD8-positive T cells (p<0.001, **Figure 1E**), followed by Nkp46/CD335-positive NK cells (p=0.002, **Figure 1G**) and PD-L1 (p=0.0262, **Figure 1F**). Scoring of COX-2 and EGFR was performed using only the semi-automatic validation method, a comparison with the manual scoring method therefore was not possible.

### Spatial Mapping of Expression Patterns

**Figure 2:**
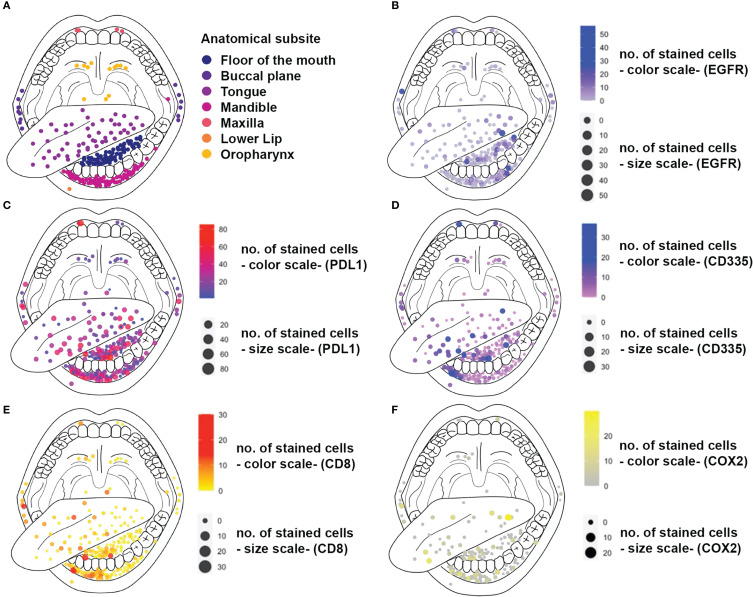
Anatograms of immunostains for different biomarkers. Diameter and color of dots represent the number of stained cells for each biomarker. The localizations of dots meet the anatomic subsites affected by the tumor. **(A)** Localization of tumor tumors within the oral cavity and the oropharynx **(B)** EGFR expression **(C)** PD-L1 expression **(D)** CD335/NK cell Infiltration **(E)** CD8 cell infiltration **(F)** COX2 Expression

The distribution of tumors and the protein expression (digital scoring) for investigated biomarkers in dependence of the affected anatomic subunit of the oral cavity are shown in **Figure 2**. A subset of tumors which were localized in the border region between the oral cavity and oropharynx (n=16; soft palate and base of the tongue) were subsumed under the category “oropharynx”. Overall, there was a dominance of tumors located in the lower section of the oral cavity with a higher frequency at the tongue, the floor of the mouth and the mandible (n=186, 83.8%; see **Table 1** and **Figure 2**).

The median expression values of the investigated biomarkers did not differ significantly between the anatomical subsites, except for PD-L1. Here, median expression values were significantly lower (p<0.05) for tumors located in the oropharynx as compared to the other subsites except for the maxilla (**Supplementary Figure 1** and **Supplementary Table 1**).

### Survival Analysis

**Figure 3:**
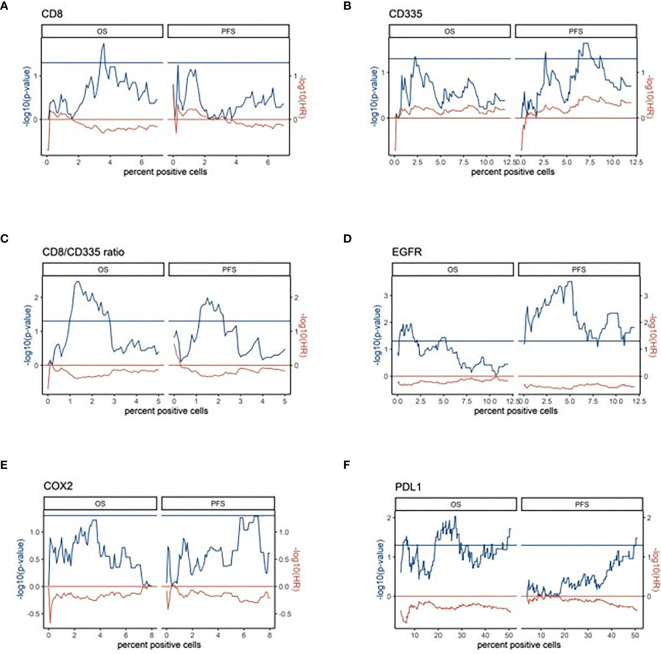
Data-driven expression cut-offs of the digital scores for survival time analysis **(A–F)**. The p-values (cox proportional hazard model, -log10) and hazard ratios (-log10) of the univariate survival analysis are plotted over the percent of positive cells in the digital scoring. The left half of the respective plots show the results for OS and the right half for PFS. Horizontal lines illustrate the p-value cutoff (p < 0.05) and the direction of association (HR=1). Here, a negative –log10 HR corresponds to a shorter OS or PFS, respectively.

**Figure 4:**
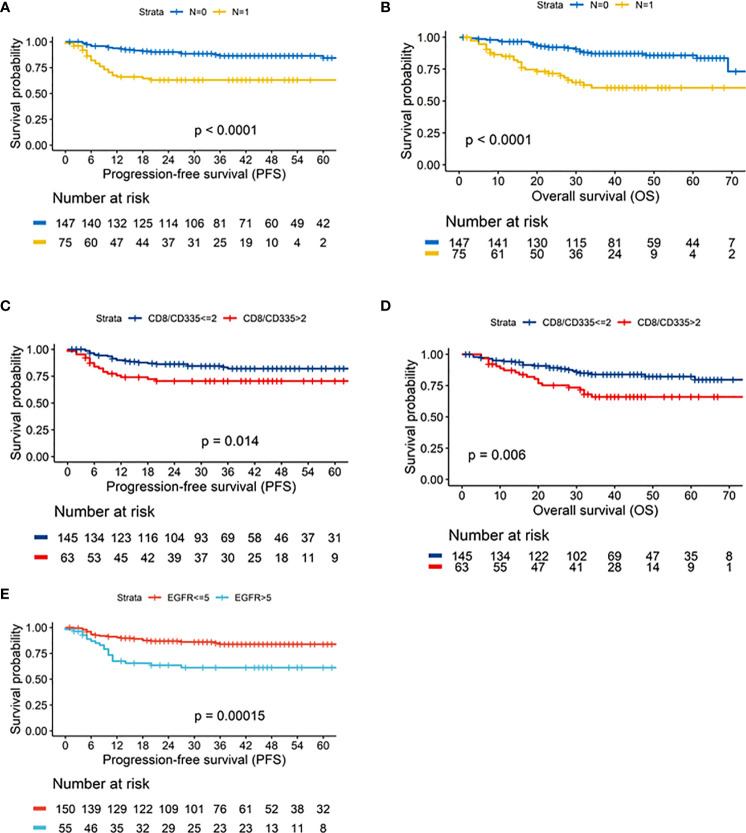
Kaplan-Meier curves depicting the results of the univariate survival analysis for: Neck node status (N0/N1) – **(A)**. Progression-free survival (PFS) – **(B)**. overall survival (OS) CD8/CD335 ratio (CD8/CD335 ≤2/>2) – **(C)**. Progression-free survival (PFS) – **(D)**. overall survival (OS) EGFR status (EGFR ≤5/>5 – **(E)**. Progression-free survival (PFS).

Assessing the clinical characteristics of the cohort revealed that the nodal status was the only parameter that was associated with OS (p=1.81E-05) or PFS (p=1.31E-05; **Figure 4**). To identify reliable expression biomarkers that could be applied by pathologist, expression values were not modelled as a continuous parameter in the survival time analysis but cut-offs were established. To this end, the distribution of p-values of the univariate cox proportional hazard model enabled to determine the robustness of cut-offs (**Figure 3**).

In the univariate survival analysis with overall survival (OS) as the endpoint, the CD8/CD335 ratio and PD-L1 but not CD8 showed a prognostic impact across a larger range of cut-offs. Both a higher CD8/CD335 ratio (ratio >2), i.e. a T cell dominance and a higher PD-L1 expression was associated with a shorter OS (-log10 hazard ratio < 0). Modelling progression free survival (PFS) as the endpoint identified the CD8/CD335 ratio and EGFR as significant biomarkers. Both markers were associated with a shorter PFS (**Figure 4**).

In the multivariate analysis of overall survival, the CD8/CD335 ratio and nodal status (N), but not PD-L1 were confirmed as independent prognostic markers (**Table 2**). The survival differences after stratification for pathological N status and CD8/CD335 ratio are illustrated in **Supplementary Figure 2B**. The best survival rates were observed for patients with N0 status and low CD8/CD335 ratio, while N+ with high CD8/CD335 ratio was associated with worse survival (p<0.001).

**Table 2:** Multivariate analysis of overall and progression-free survival.

Characteristics	Expression Cut-off	Overall survival		Progression-free survival	
		HR (95% CI)	p-value	HR (95% CI)	p-value
**N status**	**NA**	1.26 (0.65-1.87)	<0.001	1.44 (0.81-2.07)	<0.001
**CD8/CD335 ratio**	**2**	0.69 (0.09-1.29)	0.02	0.74 (0.13-1.35)	0.02
**EGFR**	**5**	–	–	1.16 (0.54-1.78)	0.002

The CD8/CD335 ratio and the EGFR expression were dichotomized according to the presented expression cut-offs. HR, hazard ratio. NA, N Status does not have a cutoff. Bold values mean for EGFR no significant impact on overall survival could be shown.

Similar results were observed for progression-free survival (PFS). Here, nodal status (N), the CD8/CD335 ratio and EGFR status were confirmed as independent prognostic markers (**Table 2**). **Supplementary Figure 2B** illustrates the survival rates after stratification for EGFR status, N status and CD8/CD335 ratio. Best survival rates were seen in patients with negative EGFR status and low CD8/CD335 ratio, irrespective of neck node status, while worst survival was observed for patients with positive EGFR status, presence of neck node metastases (N+) and high CD8/CD335 ratio (p<0.001).

## Discussion

The identification of reliable biomarkers is of critical importance for cancer research and to further individualize tumor therapy. While there have been advances in the definition of markers to prognosticate the therapeutic response to palliative medical therapy using immune checkpoint inhibition (ICI) by the evaluation of the tumor mutational burden (TMB) or the expression levels of PD-L1, there still is a lack on relevant markers for the primary disease (25–27). A plethora of publications exists on the correlation of different markers with clinical parameters, such as tumor size, neck node status or survival, including proteins or genetic material (28, 29). However, none has been established so far in clinical practice to serve as an accurate prognostic or predictive marker for primary therapy. The major goal in the primary disease stage is to stratify patients’ risks for tumor recurrence and, consequently, to allocate them to an adjusted primary and adjuvant treatment or more rigorous follow-up surveillance. Moreover, the introduction of new anti-cancer therapies is based on the identification of appropriate targets. The classical way of biomarker research is manual scoring of immunohistochemical staining to evaluate the expression levels of different proteins or to analyze quantity and distribution of tumor-associated immune cells. This technique is time-consuming and potentially hard to reproduce. This often results in numerous publications with contradictory conclusions due to differing methods of data generation, analysis and interpretation.

Hence, one of the goals of this study was to utilize digital pathology algorithms as a new standard procedure for the quantification of expression levels of potential biomarkers in oral cancer. The obtained IRS were then correlated with data obtained by manual scoring, associations with affected anatomic subsites, and prognostic significance to evaluate their conclusiveness.

Several biomarkers were chosen for this analysis including EGFR, COX-2, PD-L1, CD8-positive T cells and Nkp46/CD335-positive NK cells. The immune system has been identified as a key factor in the development of cancer and subsequently merged into the focus of cancer research. In the field of head and neck cancer, the introduction of immune checkpoint inhibition has further raised the importance of understanding the tumor-associated immune microenvironment and its potential influence on therapeutic success (11, 12, 30, 31). As response to immune checkpoint inhibition is restricted to a fraction of patients, surrogate markers for therapeutic success are needed. Here, several promising candidates have been proposed, including PD-L1 and tumor infiltration lymphocytes (TILs). PD-L1 has emerged as an independent prognostic marker in head and neck cancer patients and the application of immune-checkpoint-inhibition partly is based on PD-L1 expression levels (12, 32, 33). Several studies already reported on the high prognostic significance of TILs in various malignancies and their role as a marker for anti-tumor immune response (34–37). EGFR is an established prognostic marker, a key target in anti-cancer therapy and lately has been linked to different immune phenotypes and response to ICI treatment together with COX-2 (23, 38–40).

The primary aim of this study was to evaluate a digital pathology algorithm using the Qupath software approach to assess potential biomarkers in oral cancer. While different aspects of this method have been thoroughly described for a variety of diagnostic tasks including analysis of histological tumor samples, its utility for head and neck cancers remains to be confirmed in larger cohorts. Shaban et al. introduced a digital score for TIL abundance in OSCC investigating a cohort of 60 patients and described it as strong prognosticator for disease free survival. Moreover, they reported on the significantly higher impact of the digital TIL score in comparison to the manual score (14). de Ruiter et al. evaluated various T-cell markers in a cohort of 80 HPV-negative HNSCCs undergoing primary chemo-radiotherapy without finding relevant differences in overall and progression-free survival depending on T-cell infiltration (16). In another study, the authors used a digital pathology approach to determine PD-L1 expression and its prognostic significance in breast cancer. The authors concluded that the technique of digital pathology was effective in stratifying biomarker scores (41). In our study, the evaluation of expression levels of different proteins and the infiltrations patterns by manual scoring or semi-automatic scoring produced significantly correlated data sets. While the concordance was highest for CD8 cells, the correlations for NK cell and PD-L1 scores were weaker, possibly due to the greater variation of staining intensity and localization, especially for PD-L1.

All investigated biomarkers were analyzed regarding expression differences for distinct anatomical subsites by vector-designed anatograms depicting localization and staining intensity for each tumor and marker (**Figure 2**). Besides a dominance of tumors in the lower section of the oral cavity, there was a significant tendency towards lower median PD-L1 expression in tumors located in the oropharynx (base of the tongue and soft palate). All sixteen tumors that were subsumed under the term “oropharynx” were borderline tumors which affected the oral cavity and the oropharynx (e.g. maxilla-soft palate; tongue – base of the tongue). Those tumors were labeled as “oropharyngeal cancer” to sharpen the anatomical classifications for the analysis of spatial expression heterogeneity.

In the univariate survival analysis, several markers showed a prognostic impact on overall and progression-free survival, including PD-L1, EGFR, CD8 and NK cells. Furthermore, in the multivariate analysis, EGFR could be confirmed as independent prognostic marker for progression-free survival and the CD8/CD335 ratio for both, overall and progression-free survival. As reported before, this observation is in accordance with several other publications and confirms the validity of our data. Furthermore, this concordance strengthens the technique of digital pathology as valid method of analysis for future studies. Several authors used different immune-scores including CD8-positive T cells to identify patients at risk for adverse outcome or those who had a higher chance to profit from a more intense multi-modal treatment including radio-chemotherapy (42–45). NK cells play a vital role in anti-tumor immunity and, in contrast to T cells, are independent of MHC related activation or prior immunization and their prognostic impact on survival has been demonstrated for a variety of tumors (46–50). Their activity is mainly guided by inhibitory and activating signals *via* chemokines and blocking of inhibitory pathways has been shown to result in improved anti-tumor response (51, 52). While most of the mentioned publications on TILs in HNSCC reported on mixed cohorts of patients with primary and recurrent tumors of different subsites who mostly received different treatment modalities, our analysis focused on a large cohort of 222 patients with OSCC and primary surgical therapy. Here, we could confirm the prognostic significance of tumor-infiltrating lymphocytes (TILs) by evaluating NK cell and CD8 cell infiltration separately and in a CD8/CD335 ratio. In a multivariate analysis, the CD8/CD335 ratio was confirmed as independent prognostic factor for OS and PFS (p=0.02). The prognostic relevance of PD-L1 for oral cancer has been discussed in a plethora of studies and is supported by the data reported in this study (33).

The presented study has several limitations including the utilization of TMAs for the analyses considering the intratumor heterogeneity of several potential biomarkers such as PD-L1 (53). Further studies are warranted to evaluate the reported results using whole tissue slides and to validate the findings in a prospective setting. The translation of potential biomarkers into clinical practice is highly dependent of several factors, including prognostic value, reliability, and cost effectiveness. While singular biomarkers are prone to exhibit limitations regarding their clinical applicability, the definition of a set of several markers may synergize the strengths and compensate the weaknesses of single markers. The presented study contributed to this task by evaluating potential biomarker candidates using and validating a novel scoring approach with digital pathology on a large cohort of patients with oral cancer.

## Conclusions

The assessment of immunohistochemical staining *via* digital pathology techniques was shown to be a feasible and efficient option for objective pathological analysis. The expression levels of different proteins and concentrations of tumor-infiltrating lymphocytes were successfully evaluated. Thereby, a set of clinical and histological markers with high prognostic relevance was identified. These findings provide a valuable contribution to the establishment of digital pathology as standard procedure for the identification and validation of existing and future biomarkers with clinical relevance to further enhance risk-stratification and individualization of tumor therapy in patients suffering from oral cancer.

## Data Availability Statement

The original contributions presented in the study are included in the article/**Supplementary Material**. Further inquiries can be directed to the corresponding author.

## Ethics Statement

The studies involving human participants were reviewed and approved by Ethics Committee of the University of Heidelberg. Written informed consent for participation was not required for this study in accordance with the national legislation and the institutional requirements.

## Author Contributions

Conceptualization, JM, AM, JHe and CFr. Methodology, JM, AM, JHe, JK, CFr, SO. Formal analysis, JM, AM, SO, CFl, KZ, DJ, SF, JHe. Resources, JHo, JHe, JK, SF, DJ. Data curation, JM, AM, SO, DH, KF and KZ. Writing – original draft preparation, JM, AM, SO, DH, JHe and CFr. Writing – review and editing, CFl, CFr, DJ, SF, JHe, JHo, JK, KF, KZ and KM. visualization, JM, AM, SO and KM. All authors contributed to the article and approved the submitted version.

## Funding

JM and AM are funded by the Physician-Scientist Program of the University of Heidelberg, Faculty of Medicine, JM is funded by the “Stiftung Tumorforschung Kopf-Hals”.

## Conflict of Interest

The authors declare that the research was conducted in the absence of any commercial or financial relationships that could be construed as a potential conflict of interest.

## Publisher’s Note

All claims expressed in this article are solely those of the authors and do not necessarily represent those of their affiliated organizations, or those of the publisher, the editors and the reviewers. Any product that may be evaluated in this article, or claim that may be made by its manufacturer, is not guaranteed or endorsed by the publisher.
