# Supplementary material for: Digital Pathology Scoring of Immunohistochemical Staining Reliably Identifies Prognostic Markers and Anatomical Associations in a Large Cohort of Oral Cancers
Source: Front Oncol. 2021 Jul 29;11:712944. doi: 10.3389/fonc.2021.712944 (PMC8359738; doi:10.3389/fonc.2021.712944)
Supplement: Supplementary file 1 [file DataSheet_1.docx]

Supplementary Material

# Supplementary Data

## Supplementary Tables

**Supplementary table 1.** P-values from the pairwise comparison of PD-L1 expression in different anatomic subsites using the Wilcoxon rank sum test.

|  | **Floor of the mouth** | **Tongue** | **Mandible** | **Maxilla** | **Oropharynx** |
| --- | --- | --- | --- | --- | --- |
| **Tongue** | 0.6730 | - | - | - | - |
| **Mandible** | 0.1823 | 0.4639 | - | - | - |
| **Maxilla** | 0.4380 | 0.2597 | 0.1526 | - | - |
| **Oropharynx** | **0.0386** | **0.0125** | **0.0014** | 0.8660 | - |
| **Buccal mucosa** | 0.7833 | 0.9661 | 0.6758 | 0.5663 | 0.0885 |

1.2. Supplementary **Figures**

**
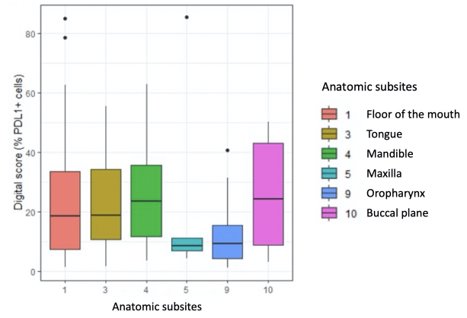
**

**Supplementary Figure 1.** Boxplots of median PD-L1 expression stratified by localization of primary tumor. Median PD-L1 expression was significantly lower in tumors of the oropharynx, compared to those of other localizations, except for tumors of the maxilla (see Supplemental Table 1).


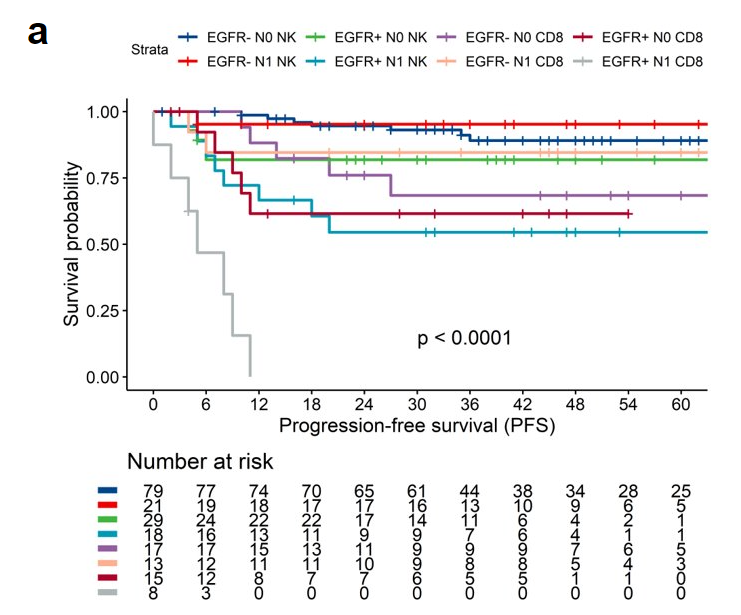

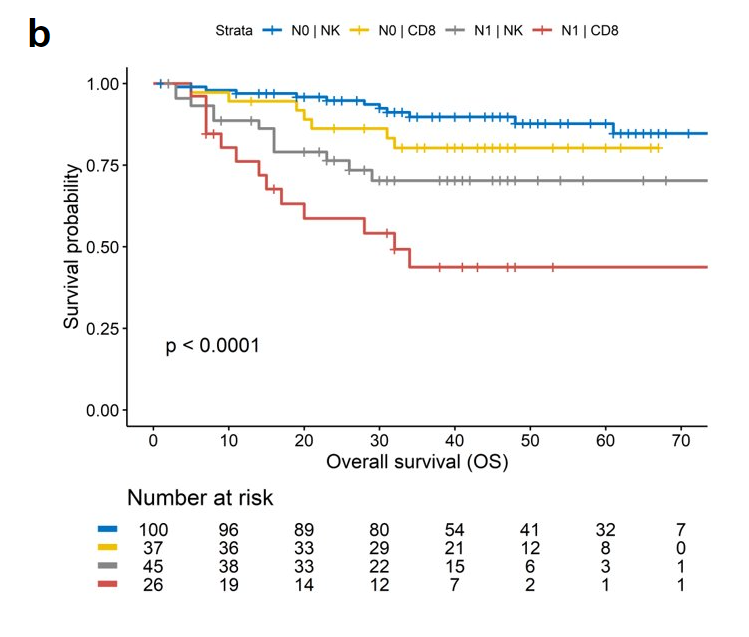


**Supplementary Figure 2.** Kaplan-Meier curves depicting the results of the multivariate regression analysis for **a**. Progression-free survival (PFS) including EGFR status (EGFR-/EGFR+), neck node status (N0/N1) and CD8/CD335 cell ratio and **b**. Overall survival (OS) including neck node status (N0/N1) and CD8/CD335 cell ratio.
